# Estimating pain visual analogue scale from health assessment questionnaire for rheumatoid arthritis with beta mixture models

**DOI:** 10.1007/s00296-025-05897-1

**Published:** 2025-06-14

**Authors:** Sean P. Gavan, Sainan Chang, Felice Rivellese, Zoë Ide, Michael Stadler, Katherine Payne, Darren Plant, Anne Barton, Costantino Pitzalis

**Affiliations:** 1 Manchester Centre for Health Economics, Division of Population Health, Health Services Research and Primary Care, School of Health Sciences, Faculty of Biology, Medicine and Health, The University of Manchester, Manchester, M13 9PL UK; 2 Centre for Experimental Medicine and Rheumatology, Queen Mary University of London, London, UK; 3 Department of Rheumatology, Mile End Hospital, Barts Health NHS Trust and NIHR Barts Biomedical Research Centre, London, UK; 4 Patient Researcher, The University of Manchester, Manchester, UK; 5 Versus Arthritis Centre for Genetics and Genomics, Centre for Musculoskeletal Research, Division of Musculoskeletal and Dermatological Sciences, School of Biological Sciences, Faculty of Biology, Medicine and Health, The University of Manchester, Manchester Academic Health Science Centre, Manchester, UK; 6 NIHR Manchester Biomedical Research Centre, Manchester University NHS Foundation Trust, Manchester Academic Health Science Centre, Manchester, UK

**Keywords:** Beta mixture model, Health assessment questionnaire, Mapping, Patient-reported outcomes, Pain, Rheumatoid arthritis, Visual analogue scale

## Abstract

To map from the health assessment questionnaire disability index (HAQ) to the pain visual analogue scale (VAS) for people with rheumatoid arthritis. The estimation sample comprised adults with rheumatoid arthritis and inadequate response to tumour necrosis factor-α inhibitors in a multicentre phase 4 randomised controlled trial. Beta mixture models were estimated with combinations of HAQ and its square, age and sex as independent variables. Bayesian Information Criteria informed the number of components. Model performance (root mean squared error; mean absolute error; pseudo-R^2^) was estimated by k-fold cross validation. Graphs illustrated mean observed and predicted pain VAS, and cumulative distribution of observed and simulated pain VAS values. For face validity, a probabilistic analysis simulated 5000 pain VAS values at four HAQ scores. For external validation, the performance of the preferred specification was assessed using the Rheumatoid Arthritis Medication Study cohort. There were 1055 observations from 158 participants in the estimation sample (mean age: 55.8; 81% female; mean HAQ: 1.72). The preferred specification was a two-component beta mixture model (probability variables: HAQ, age, sex; main regression variable: HAQ). Visual plots illustrated good fit across the HAQ distribution, and a similar cumulative distribution of observed and predicted pain VAS values. Probabilistic analysis demonstrated that the preferred specification handled uncertainty appropriately. External validation demonstrated that the preferred specification performed well in an independent dataset. Beta mixture models provide accurate non-linear estimates of pain VAS from HAQ scores to support evidence synthesis and resource allocation decision-making for people with rheumatoid arthritis.

**Supplementary Information:**

The online version contains supplementary material available at 10.1007/s00296-025-05897-1.

## Introduction

Rheumatoid arthritis is a common lifelong autoimmune condition that affects around 17.6 million people globally [1]. Symptoms include inflammation, swelling or tenderness in the joints, and gradual irreversible joint damage if left untreated [2]. Patient-reported outcomes are vital to improve care by emphasising how people with rheumatoid arthritis perceive their own health status [3]. Increased pain and reduced functional ability are two important patient-reported outcomes that reflect the impact of ongoing active disease [4]. Reliable estimates of pain and functional ability both contribute to a better understanding of health-related quality of life [5]. However, robust estimates of patient-reported pain across the extent of functional ability are not currently available, which reduces the evidence available to support healthcare decision-making for rheumatoid arthritis.

The Health Assessment Questionnaire Disability Index (HAQ) is a patient-reported outcome that measures functional ability (higher scores indicate worse functional ability) [6]. A visual analogue scale (VAS) is used to measure patient-reported pain (higher scores indicate worse pain) [7]. Previous studies have demonstrated a positive correlation between these two outcome measures [8, 9]. However, this relationship is not linear as the rate of increase in pain VAS values appears to decline for higher HAQ scores [10].

Linear regression has been used by many earlier studies to estimate the association between HAQ scores and pain VAS values for people with rheumatoid arthritis [11, 12]. Linear regression in this context is limited in three specific ways. First, by imposing a linear association, the corresponding pain VAS values are likely to be overestimated at the upper tail of the HAQ distribution. Second, studies that reported parameter estimates without the corresponding intercept term cannot be used to predict pain VAS values at the individual-level. Finally, linear regression can lead to implausible predicted pain VAS values because it does not account for the bounded nature of the VAS scale [13].

Recent advances in the mapping literature have demonstrated the importance of using mixture models to better reflect the complexities of patient-reported outcomes (including bounded outcome variables, non-linearities, and mass points) [13]. Mapping methods have predominantly been used to estimate preference-based health utility values, but the same techniques can also be used to improve estimates between non-preference-based patient-reported outcome measures [14]. For patient-reported VAS outcomes, in particular, beta mixture models offer advantages over linear regression [15]. For example, the multinomial functional form can predict the likelihood of mass point observations at the tails of a VAS distribution [16]. Mixtures of beta models can capture non-linearities between the boundaries of the VAS distribution informed by patient-level characteristics [16]. The approach can reveal groups of distinct VAS distributions that are latent within a sample, and use patient-level characteristics to estimate the likelihood of belonging to each group. No study to date has used beta mixture models to estimate pain VAS values, which presents an opportunity to demonstrate how this method can improve estimates of pain for people with rheumatic conditions. Building on these beta mixture model methods, the aim of this study was to map from the HAQ to the pain VAS for people with rheumatoid arthritis.

## Materials and methods

A mapping study was performed to estimate pain VAS values from the HAQ for people with rheumatoid arthritis. The study was reported according to the Mapping onto Preference based measures and reporting Standards (MAPS) statement [17].

### Data source

The estimation sample comprised the pooled baseline and follow-up patient-level observations from the phase 4 R4RA randomised controlled trial (ISRCTN ID: ISRCTN97443826; EudraCT ID: 2012–002535-28) [18]. The R4RA trial estimated the effectiveness of a synovial tissue biopsy to inform treatment selection with rituximab or tocilizumab for people with rheumatoid arthritis. Participants were recruited from 19 centres across the UK, Belgium, Italy, Portugal, and Spain. The inclusion criteria were adults (18 years or older) who met the 2010 ACR/EULAR Rheumatoid Arthritis classification criteria [19], had inadequate response to a tumour necrosis factor-α inhibitor, were eligible to receive rituximab according to recommendations by the National institute for Health and Care Excellence, were receiving a stable dose of methotrexate for at least 4 weeks before their biopsy, were capable of providing informed consent, and were willing to participate in the trial [18]. The published trial report provides full details of the R4RA trial design and recruitment strategy; the exclusion criteria can also be found in the first table of the trial report’s open access supplementary material [18].

### Instruments

For this mapping study, the HAQ was the source instrument and the pain VAS was the target instrument [6, 7]. The HAQ is a 20-item patient-reported outcome which asks questions about functional ability across eight domains (dressing, arising, eating, walking, hygiene, reach, grip, and common daily activities) [6]. Each domain has at least two items. Responses to each item have four levels: 0 (without any difficulty); 1 (with some difficulty); 2 (with much difficulty); and 3 (unable to do). The total HAQ score (range: 0–3) is calculated as the mean of the highest level response within each domain. HAQ was collected at eight timepoints: one pre-baseline timepoint; baseline; 12 weeks; 24 weeks; 36 weeks; 48 weeks; 72 weeks; and 96 weeks. The pain VAS is a 100 mm VAS which asked participants to self-report their pain on a scale of 0 (no pain) to 100 (worst possible pain) [7]. The corresponding pain VAS observations were obtained from the same follow-up timepoints as the HAQ observations.

### Descriptive analysis

The estimation sample was summarised by their mean values (age; proportion female and male; disease duration; HAQ; pain VAS; disease activity index 28-joint count with C-reactive protein (DAS28-CRP) and erythrocyte sedimentation rate (DAS28-ESR) [20]; the Clinical Disease Activity Index (CDAI) [21]; previous methotrexate use; and the number of previous biologic treatments. A Spearmen’s rank correlation coefficient was estimated to establish the conceptual overlap between the HAQ and pain VAS. This correlation was hypothesised to be positive because worse functional ability (higher HAQ) was assumed to be associated with greater self-reported pain (higher pain VAS). The strength of the correlation was determined by the absolute value of the correlation coefficient (very weak: 0.00–0.19; weak: 0.20–0.39; moderate: 0.40–0.59; strong: 0.60–0.79; very strong: 0.80–1.00) [22].

### Model development

A series of beta mixture models were estimated, which are appropriate for handling dependent variables that measure responses between zero to one and have a high likelihood of observations at the boundary of the measurement scale [16]. The observed pain VAS was first divided by 100 to rescale to a 0 to 1 interval. The estimated models comprised a multinomial logistic regression for observations at the boundaries of the pain VAS distribution and a beta distribution for observations within the bounded interval. Four combinations of independent variables (HAQ, HAQ^2^, age, male sex) were used to estimate pain VAS values. To support interpretation, these combinations of independent variables were coded with uppercase letters (A–D) according to the specifications in Table 1. All combinations included demographic variables to mitigate the risk of bias due to misspecification following recommendations in the mapping literature [14]. The analysis estimated up to three component beta distributions for each model specification. Components were added sequentially by using the estimated parameters from the preceding regression as the starting values for the subsequent regression. The lowest Bayesian Information Criterion (BIC) value was used to determine the number of components for each specification [23]. The probability of being at the boundary of the pain VAS distribution and component membership was also estimated by the four combinations of independent variables; these specifications were coded using lowercase letters (a to d) when referring to probability estimates (Table 1). Standard errors were clustered at the individual-level to handle within-person correlations in observations. Beta mixture models were fit using the betamix command in Stata version 16.1 (College Station, TX: StataCorp LLC) [16].

**Table 1:** Independent variable specification for mapping regression models

Main regression variables	
Code (1st Digit)	Variables
A	HAQ
B	HAQ, Age
C	HAQ, Age, Male
D	HAQ, HAQ^2^, Age, Male

Component probability variables	
Code (2nd Digit)	Variables
a	HAQ
b	HAQ, Age
c	HAQ, Age, Male
d	HAQ, HAQ^2^, Age, Male

Number of components	
Code (3rd Digit)	Components (n)
1	1 component
2	2 components
3	3 components

Three-digit code identifies each mapping regression model according to its main regression variables, component probability variables, and number of components

### Model performance

The sixteen specifications with the lowest BIC values (one specification per combination of independent variables predicting pain VAS and probability of component membership) were taken forward for further assessment. K-fold cross validation was performed by splitting the sample into k groups at random [24]. The models were re-estimated using the k-1 groups and the remaining group was used as the validation sample (k = 4 replications performed). Model performance was assessed by the root mean squared error (RMSE), mean absolute error (MAE), and pseudo-R^2^ averaged across the K-fold validation samples. Better model performance corresponded with lower values for RMSE and MAE, and higher pseudo-R^2^ values, respectively.

Visual plots were produced to demonstrate accuracy of the model specifications across the pain VAS and HAQ distributions [13]. First, mean estimated pain VAS values were presented alongside mean observed pain VAS values (grouped by each legitimate HAQ value) to illustrate whether predictions were accurate across the HAQ distribution [13]. Second, 1000 pain VAS values were simulated for each participant to determine how each regression model handled uncertainty across the predicted distribution [13]. Simulations comprised random sampling from a beta distribution characterised by the estimated mean and variance and a uniform distribution to estimate component membership (for specifications with more than one component). The cumulative distribution of observed pain VAS values was presented alongside the cumulative distribution of simulated pain VAS values for each model specification. The preferred specification was determined by the performance statistics, the visual plots, and model parsimony. To assess the face validity of the preferred specification within a probabilistic analysis, the distribution of 5000 simulated pain VAS values were reported for a hypothetical cohort of individuals (mean age: 60 years; 50% female) at three different HAQ scores (0–3) [25]. The probabilistic analysis was achieved by Monte Carlo simulation which was parametrised by the Cholesky decomposition of the covariance matrix for the preferred mapping regression specification.

### External validation

External validation of the preferred mapping model was performed using individual participant data from the Rheumatoid Arthritis Medication Study (RAMS) [26]. The RAMS is a UK-based multicentre longitudinal cohort of people with rheumatoid arthritis and undifferentiated polyarthritis starting methotrexate for the first time. The external validation dataset comprised pooled baseline, 6-month, and 12-month observations of age, sex, HAQ, and pain VAS. The preferred mapping model was used to estimate the corresponding pain VAS value for each participant in the validation dataset. The mean observed and estimated pain VAS values were then reported visually for each HAQ score to assess performance in the external dataset across the HAQ distribution. To explore uncertainty, 1000 pain VAS values were simulated for each participant in the validation dataset using the regression output. The cumulative distributions of the simulated values and the observed pain VAS values were then compared.

## Results

**Fig. 1:**
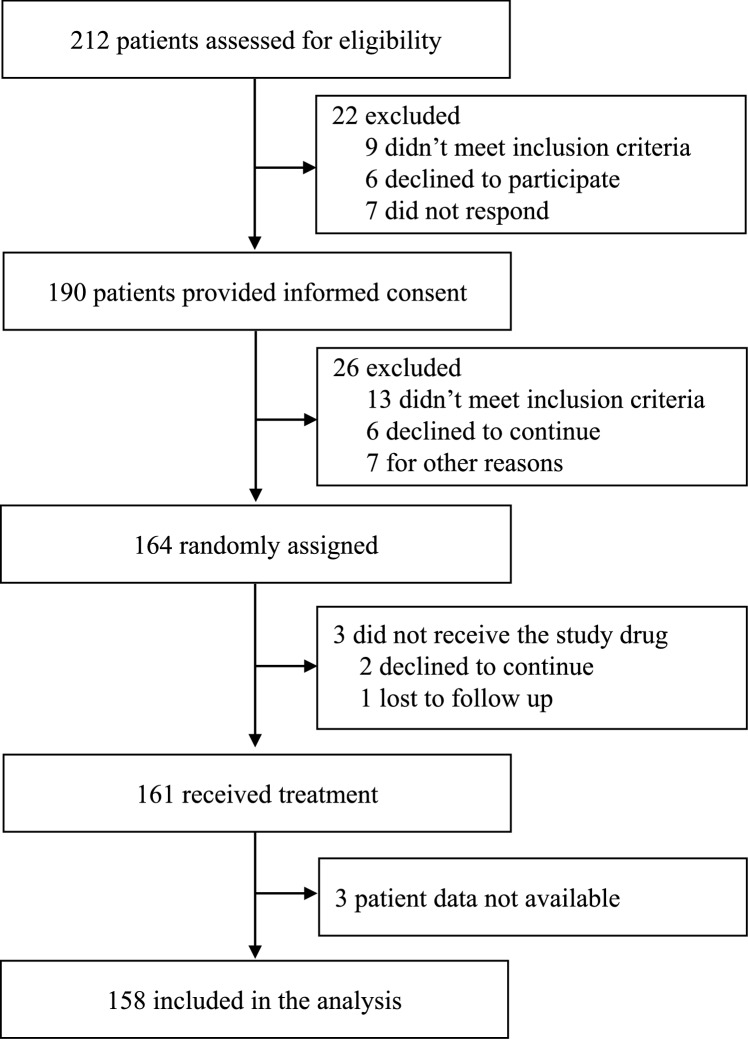
Participant inclusion flow diagram

The estimation sample comprised 1055 observations from 158 participants. Figure 1 illustrates the participant inclusion process. Table 2 reports the baseline descriptive statistics of the participants. The mean age was 55.8 years and 19% were male. The mean pain VAS was 64.7 and the mean HAQ score was 1.72. The mean DAS-28 observations exceeded 5.1 and the mean CDAI exceeded 22 which indicated that the participants had ongoing high disease activity. The Spearman’s rank correlation coefficient was 0.575 which indicated a positive moderate correlation between the reported pain VAS values and HAQ scores.

**Table 2:** Baseline descriptive characteristics of the estimation sample

Variable	All participants (n = 158)
Sex	
Female, n (%)	128 (81%)
Male, n (%)	30 (19%)
Age, years (SD)	55.8 (12.9)
Pain VAS (SD)	64.7 (24.5)
HAQ (SD)	1.72 (0.64)
DAS-28 ESR (SD)	5.82 (1.22)
DAS-28 CRP (SD)	5.32 (1.19)
CDAI (SD)	32.4 (13.8)
Disease duration (SD)	12.6 (10.7)
Previous methotrexate use	158 (100%)
Previous biologic treatment	
One	114 (72%)
Two	35 (22%)
Three or more	9 (6%)

*CDAI* Clinical Disease Activity Index, *CRP* C-reactive protein, *DAS-28* Disease Activity Score-28 joint count, *ESR* Erythrocyte sedimentation rate, *HAQ* Health Assessment Questionnaire – Disability Index, *SD* standard deviation, *VAS* visual analogue scale

The estimated BIC values for the alternative model specifications are reported in Supplementary Table S1 (Online Resource 1). According to these BIC values, models with three components always decreased fit with the observed data compared with models that had fewer components. Only one specification performed better with two components (specification: Ac2); the estimated BIC for the fifteen remaining specifications was lowest for models comprising one component.

The estimated mean pain VAS and the mean K-fold cross validation performance statistics for models that had the lowest BIC values across the sixteen alternative specifications of independent variables are reported in Supplementary Table S2 (Online Resource 1). All estimated models produced similar performance statistics across the full sample of observations. The estimated RMSE ranged between 0.238 and 0.241. The estimated MAE ranged between 0.199 and 0.201. The estimated pseudo-R^2^ ranged between 0.325 and 0.341. Therefore, it was not possible to select a preferred regression specification from the performance statistics alone because of their similarity in values.

The figure plotting the observed and predicted pain VAS values across the range of HAQ scores for all sixteen regression specifications is reported in Supplementary Figure S1 (Online Resource 1). The observed data show a positive association between HAQ and pain VAS values. All models predicted similar mean pain VAS values across the middle of the HAQ distribution. The majority of models (13 out of 16) overestimated pain VAS values at the lower end of the HAQ distribution. Models with more complex specifications (Specification D) and with more than one component in the mixture model appeared to fit the observed data better across the whole distribution of HAQ scores.

The cumulative distributions of the observed and simulated pain VAS values for each regression specification are reported in Supplementary Figure S2 (Online Resource 1). For all regression specifications, the cumulative distribution of simulated pain VAS values (dashed line) followed the distribution of the observed pain VAS values (solid line) closely across the middle and upper regions of the distribution. The cumulative distributions were closest for the regression specification that comprised a mixture model with two components.

**Fig. 2:**
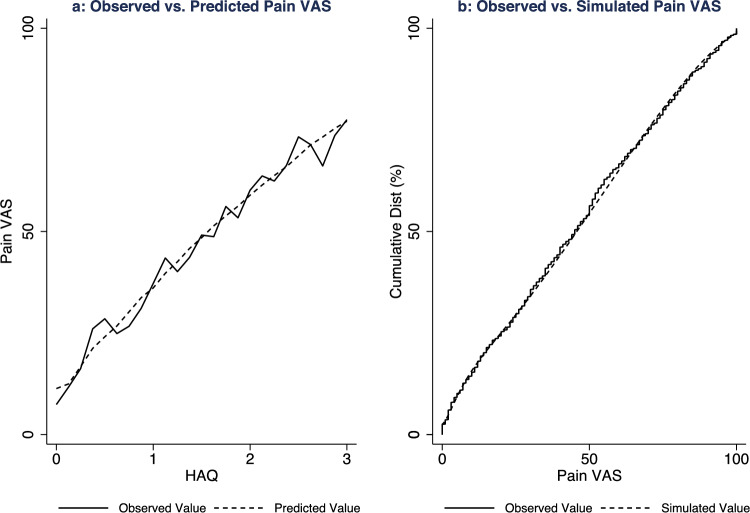
Visual plots of the preferred mapping regression model specification: development data. **a**: Mean observed and predicted pain visual analogue scale values across the Health Assessment Questionnaire Disability Index distribution; **b**: cumulative distribution of the observed and simulated pain visual analogue scale values from the preferred mapping regression model specification (Ac2)

On balance, specification Ac2 was the preferred model to estimate pain VAS values from HAQ scores by accounting for performance statistics, visual inspection of the graphed data, and model parsimony. This specification is a two-component beta mixture model which used HAQ, age, and male sex to estimate the probability of component membership or boundary pain VAS values, and HAQ alone to estimate the pain VAS value within each component. Figure 2 illustrates the (a) mean observed and predicted pain VAS values across the HAQ distribution and (b) cumulative distribution of the observed and simulated pain VAS values for this preferred regression specification.

**Fig. 3:**
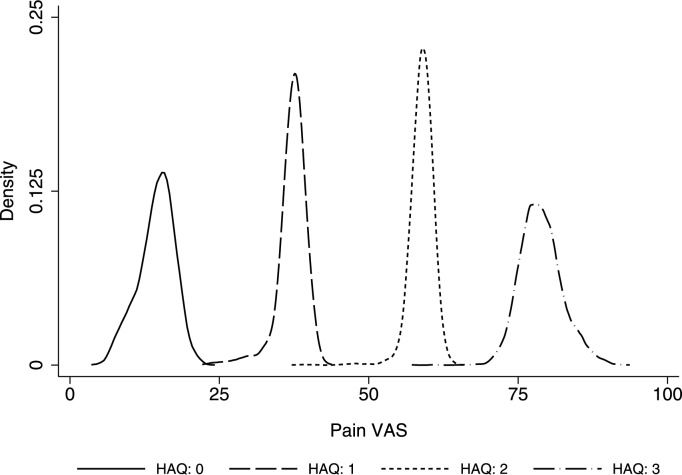
Density of simulated pain VAS values by HAQ score (n = 5,000 simulations). *HAQ* Health Assessment Questionnaire Disability Index, *VAS* visual analogue scale

To demonstrate the face validity of the preferred mapping model, Fig. 3 reports the distributions of 5000 simulated pain VAS values at four HAQ scores (0–3) for a hypothetical cohort of people with rheumatoid arthritis (mean age: 60-years; proportion female: 50%). The simulated values all fall within the bounded range of the pain VAS. The mean simulated pain VAS values increased as HAQ scores increased. The width of the simulated distributions illustrates that the extent of parameter uncertainty was greatest at the tails of the HAQ scale due to their being fewer observations at these boundary points.

**Fig. 4:**
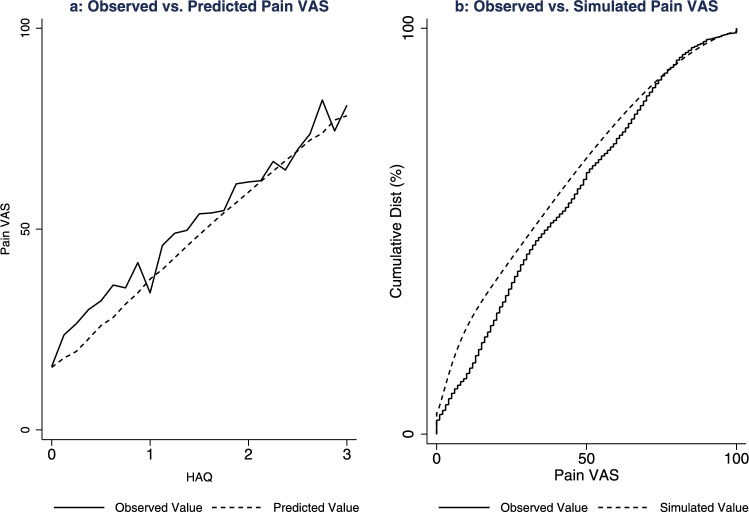
Visual plots of the preferred mapping regression model specification: validation data. **a**: Mean observed and predicted pain visual analogue scale values across the Health Assessment Questionnaire Disability Index distribution; **b**: cumulative distribution of the observed and simulated pain visual analogue scale values from the preferred mapping regression model specification (Ac2)

The external validation dataset comprised 3973 observations from 1758 participants in the RAMS cohort. Figure 4 illustrates the corresponding model performance plots for the external validation sample. The preferred mapping specification (Ac2) performed well across the HAQ distribution in this external validation sample. At lower HAQ scores, the predicted pain VAS values were underestimated by a small magnitude (approximately 5 units). This effect contributed to the cumulative distribution of simulated pain VAS values laying above the observed pain VAS values at the lower end of the distribution (Fig. 4b).

To support the implementation of the mapping model, the Online Resource 2 includes the estimated regression parameters, covariance matrix, and Cholesky decomposition to handle parameter uncertainty within future studies. In addition, Online Resource 3 includes a standalone calculator for this preferred mapping model in a Microsoft Excel file so that analysts can estimate pain VAS values rapidly by inputting a patient profile defined by HAQ, age, and sex.

## Discussion

This study used recent advances in mapping methods to estimate pain VAS values from HAQ scores for people with rheumatoid arthritis. The preferred model specification was a two-component beta mixture model comprising HAQ, age, and sex as independent variables. This preferred model specification fit the observed data well across the distribution of HAQ scores and handled parameter uncertainty effectively. The findings from this study will be valuable to support decision-making for people with rheumatoid arthritis by providing reliable estimates of expected pain VAS values when these observations are not available.

Pain and functional ability are two important dimensions known to drive quality of life and outcomes from ongoing treatment. For example, negative illness perception is greater for people with rheumatic conditions who report worse pain and functional ability, which emphasises the importance of accounting for these dimensions during routine management [27]. Self-reported pain is also associated with worse patient global assessment scores [28]. Heterogeneity between individuals in how they perceive pain is also likely, with some individuals tending to report worse pain due to ruminating or feeling helpless, resulting in lower health-related quality of life compared with individuals who do not share these traits [29, 30].

A positive correlation between high HAQ scores and worse self-reported pain outcomes has been found by previous studies [11, 12]. The estimated positive moderate correlation in the descriptive analysis was appropriate from a clinical perspective because the HAQ measures constructs reflecting functional ability that are independent of pain [6, 7]. From a statistical perspective, the magnitude of this correlation was sufficient to support a mapping analysis [31]. Previous studies have used linear regression to estimate the statistical association between HAQ and pain. For example, Yoshii et al. [12] found that a unit increase in HAQ was associated with a 19.10 (95% confidence interval: 15.3–22.9) increase in pain VAS values from a univariate linear regression. Similarly, Sarzi-Puttini. et al. [11] reported that a unit increase in HAQ was associated with a 6.47 (95% confidence interval: 1.56–11.38) increase in pain VAS values from a multivariable linear regression. However, linear regression is not likely to be appropriate for predicting pain VAS values from HAQ scores due to the bounded nature of the VAS scale, the presence of mass points at the tails of the distribution, and the potential for non-linearities in patient-reported pain outcomes. Mapping studies that use mixture models are becoming more common to improve the accuracy of predicted patient-reported outcomes from condition-specific source instruments because they can overcome the limitations faced by linear regression [13]. By building on these earlier published findings, the beta mixture model regression is a key strength of the present study because of its ability to handle the unique distribution of VAS scales appropriately and deliver outputs that meet the needs of healthcare decision-makers.

To use the outputs from this mapping study in the future, analysts will require a dataset comprising observations for HAQ scores and demographics (age and sex). These observations can be sourced from individual participant data within randomised controlled trials or observational cohorts. Alternatively, if individual participant data are not available, the dataset can be populated with mean HAQ and demographic data from a sample of participants reported within published studies. The mapping model can then be applied to this dataset to estimate plausible mean pain VAS values for each observation. If there are relatively few observations, then the online calculator in Online Resource 3 may be the best approach to estimate pain VAS values by manually entering a profile defined by HAQ, age and sex. By contrast, if the sample is sufficiently large to preclude this manual approach, then the corresponding pain VAS values can be estimated using statistical software such as R or Stata to automate the process. To achieve this, analysts will require the estimated regression coefficients and formula to calculate the pain VAS values which can be found in the ‘Formula’ tab of Online Resource 3. Propagating uncertainty in the predicted mean pain VAS values can be achieved using the Cholesky decomposition of the covariance matrix (Online Resource 2) as is standard for parameters estimated by multivariable regression [32].

The HAQ is an important patient-reported outcome used by model-based health economic evaluations for rheumatoid arthritis to characterise the trajectory of improvements and progression of disease over patients’ lifetimes [33]. The predominant measure of health benefit in these health economic evaluations is the quality-adjusted life year (QALY), underpinned by preference-based instruments such as the EQ-5D (3L or 5L versions) to measure health utility values [34, 35]. Both the HAQ and pain VAS overlap with important quality of life dimensions captured by the EQ-5D instruments (specifically: mobility, self-care, usual activities, and pain) [5]. Hernández Alava developed a mapping model to demonstrate how EQ-5D-3L utility values can be estimated from both patient-reported HAQ scores and pain VAS values for people with rheumatoid arthritis [5]. This mapping model has been pivotal for subsequent health economic evaluations in rheumatoid arthritis, and most notably informed QALY calculations for health technology assessment decisions by the National Institute for Health and Care Excellence [10]. However, analysts may not have access to pain VAS data which will prevent them from calculating QALYs using the approach by Hernández Alava et al. [5]. Therefore, to resolve this important evidence gap, the estimated pain VAS outputs from the present study can be used in conjunction with the mapping model by Hernández Alava et al. [5]. to estimate QALYs within future model-based health economic evaluations for rheumatoid arthritis.

One limitation of this study was that there were relatively fewer observations at the tails of the HAQ distribution within the estimation sample. As a consequence, parameter uncertainty in the estimated pain VAS values will likely be higher in these regions. However, this parameter uncertainty can be handled by the accompanying covariance matrix and the simulated expected values were found to have face validity. Analysts should be cautious about the magnitude of the expected predicted pain VAS values at the extremes of the HAQ distribution (0 or 3). However, the risk of this affecting decision-making is likely to be limited because few patients in routine clinical settings report these extreme values. For example, from a sample of 16,011 people with rheumatoid arthritis in the US National Data Bank for Rheumatic Diseases, only 64 people (0.004% of the participants) reported a HAQ score of 3 [5]. A second limitation was the estimation sample comprised participants who met the inclusion criteria for the R4RA trial [18], who may not be representative of people with rheumatoid arthritis in a routine clinical setting. The statistical association between the HAQ and pain VAS may be different for people who do not share the same disease characteristics of participants in this trial setting. However, the external validation with an independent observational early arthritis cohort showed that the preferred specification performed well in this sample of people who shared different disease characteristics to those within the R4RA trial. A third limitation was that the empirical specifications excluded variables such as disease activity and disease duration, which may both affect pain VAS values independently of the HAQ. Mixture models with many independent variables require large samples to converge, which precluded these variables from being included in the present study. As a result, the recent mapping literature tends to favour well-fitting but parsimonious mixture model specifications. Predicting pain VAS values from a multivariable specification of different patient-reported and clinical variables is a distinct empirical question from the aim of this study, requiring a much larger sample size, and would be a valuable topic for future research.

Future research could use beta mixture models to improve estimates of the condition-specific association between patient-reported HAQ scores and pain VAS values for other long-term conditions such as systemic lupus erythematosus and psoriatic arthritis. More generally, beta mixture models can be used to improve predicted values for other patient-reported VAS outcomes, such as global VAS assessments of health, by accounting for the bounded nature of these scales and mass points in the distribution. Where pain VAS values are not available in published data, future research can also use the findings from this study to estimate these pain VAS values based on the reported HAQ and demographic data to align with core outcome data recommendations for rheumatoid arthritis [36, 37]. Finally, future trial-based and model-based health economic evaluations in rheumatoid arthritis can use the estimates from this study to improve the evidence available for estimating health state utility values that require pain VAS data.

## Conclusion

Patient-reported outcomes that reflect pain and functional ability can help to establish the value of care by capturing how people with rheumatoid arthritis perceive improvements in these important health benefits. Healthcare decision-makers and analysts need to understand the association between pain and functional ability to improve how these outcomes are characterised over time. This mapping study will support decision-making by improving estimates of patient-reported pain outcomes in scenarios where these data are unavailable. People with rheumatoid arthritis will ultimately benefit from this study by strengthening the evidence base available to support healthcare resource allocation decisions for individuals in this population with ongoing active disease.

## Supplementary Information

Below is the link to the electronic supplementary material.

Supplementary file1 (PDF 276 KB)

Supplementary file2 (XLSX 19 KB)

Supplementary file3 (XLSX 13 KB)

## Data Availability

Data for this study cannot be shared openly because participants did not provide informed consent for this during ethical approval. Access to anonymised data from the R4RA trial (10.1016/s0140-6736(20)32341-2) and the Rheumatoid Arthritis Medication Study cohort (10.1186/s13075-018-1645-5) may be granted following review with the relevant study coordinators. Contact details for the study coordinators can be found in their respective publications.
