# Supplementary material for: Estimating pain visual analogue scale from health assessment questionnaire for rheumatoid arthritis with beta mixture models
Source: Rheumatol Int. 2025 Jun 14;45(7):154. doi: 10.1007/s00296-025-05897-1 (PMC12167326; doi:10.1007/s00296-025-05897-1)

Estimating Pain Visual Analogue Scale from Health Assessment Questionnaire for Rheumatoid Arthritis with Beta Mixture Models

Supplementary Appendix

Contents

Page 2: Table S1. Estimated Bayesian Information Criteria values for candidate mapping regression models

Page 3: Table S2. K-fold cross validation performance statistics

Page 4: Figure S1. Mean observed and predicted pain VAS values across the HAQ distribution for each mapping regression specification

Page 5: Figure S2. Cumulative distribution of observed and simulated pain VAS values for each mapping regression specification

Table S1. Estimated Bayesian Information Criteria values for candidate mapping regression models

Mapping Specification ID	BIC	Mapping Specification ID	BIC
**Aa1**	**-20.290**	**Ca1**	**-18.956**
Aa2	-11.765	Ca2	-0.926
Aa3	-7.783	Ca3	21.828
**Ab1**	**-11.873**	**Cb1**	**-10.539**
Ab2	-6.825	Cb2	NC
Ab3	NC	Cb3	NC
Ac1	-4.228	**Cc1**	**-2.894**
**Ac2**	**-7.402**	Cc2	12.832
Ac3	9.740	Cc3	NC
**Ad1**	**8.743**	**Cd1**	**10.077**
Ad2	NC	Cd2	43.044
Ad3	NC	Cd3	76.460
**Ba1**	**-13.777**	**Da1**	**-14.511**
Ba2	0.589	Da2	11.320
Ba3	17.065	Da3	47.324
**Bb1**	**-5.360**	**Db1**	**-6.093**
Bb2	15.577	Db2	29.814
Bb3	27.194	Db3	NC
**Bc1**	**2.285**	**Dc1**	**1.551**
Bc2	NC	Dc2	39.551
Bc3	NC	Dc3	68.039
**Bd1**	**15.256**	**Dd1**	**14.523**
Bd2	26.177	Dd2	61.766
Bd3	NC	Dd3	NC

Mapping specification ID refers to the combination of independent variables and number of components described in Table 1. Bold values highlight the lowest Bayesian Information Criteria within each family of regression specifications. NC indicates that the maximum likelihood estimation did not achieve convergence.

Table S2. K-fold cross validation performance statistics

Mapping Specification ID	Mean Predicted Pain VAS/100	Root Mean Square Error	Mean Absolute Error	Pseudo-R^2
Aa1	0.456	0.240	0.201	0.328
Ab1	0.456	0.240	0.200	0.332
Ac2	0.458	0.239	0.199	0.327
Ad1	0.456	0.240	0.200	0.335
Ba1	0.456	0.240	0.200	0.330
Bb1	0.456	0.241	0.200	0.328
Bc1	0.456	0.240	0.200	0.325
Bd1	0.456	0.240	0.200	0.332
Ca1	0.456	0.239	0.199	0.336
Cb1	0.456	0.240	0.199	0.340
Cc1	0.457	0.239	0.200	0.335
Cd1	0.457	0.239	0.199	0.336
Da1	0.457	0.239	0.201	0.339
Db1	0.457	0.238	0.199	0.335
Dc1	0.457	0.239	0.200	0.337
Dd1	0.457	0.240	0.199	0.341

Abbreviation: VAS, visual analogue scale. Mapping specification ID refers to the combination of independent variables and number of components described in Table 1.

Figure S1. Mean observed and predicted pain VAS values across the HAQ distribution for each mapping regression specification

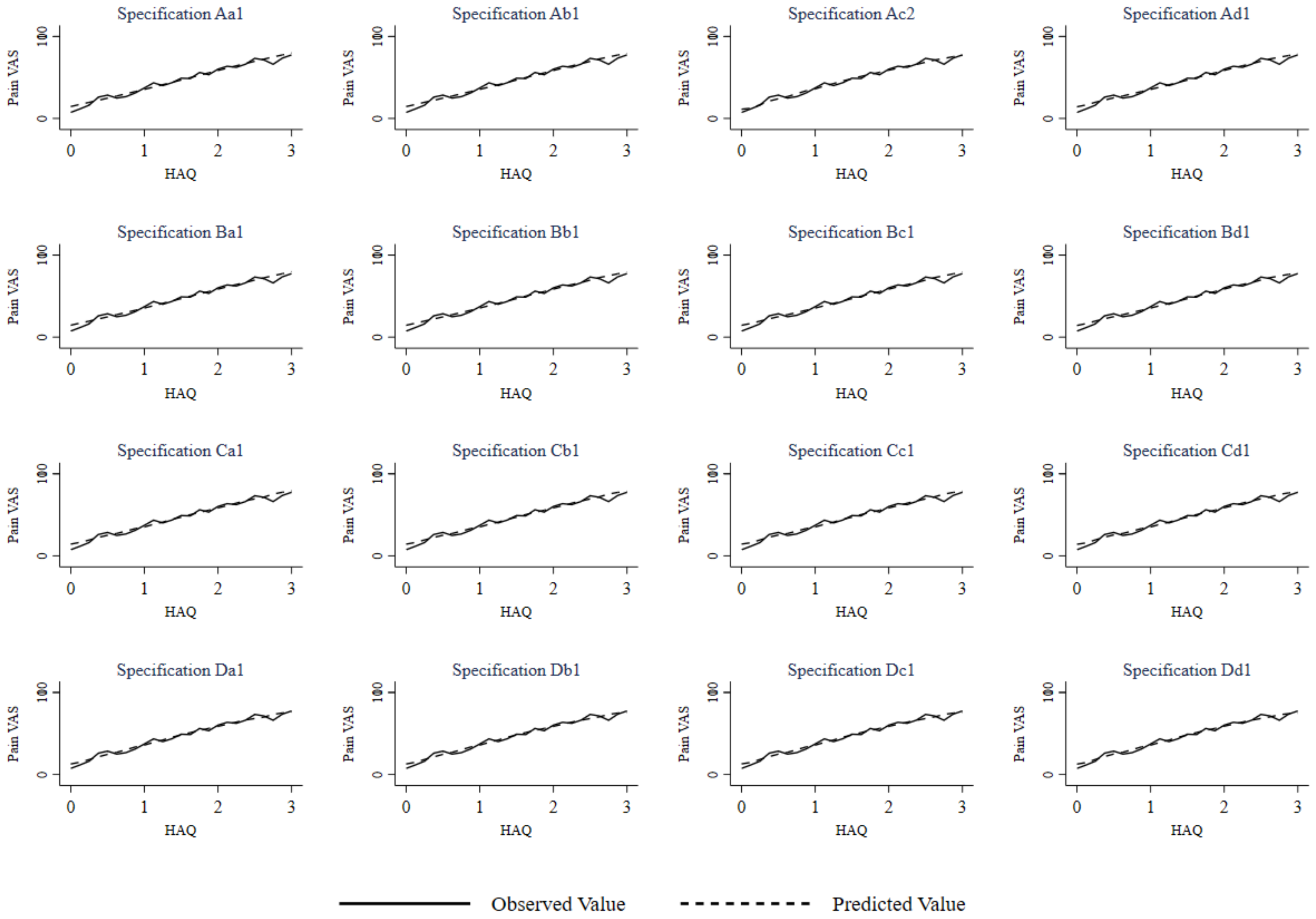

Figure S2. Cumulative distribution of observed and simulated pain VAS values for each mapping regression specification

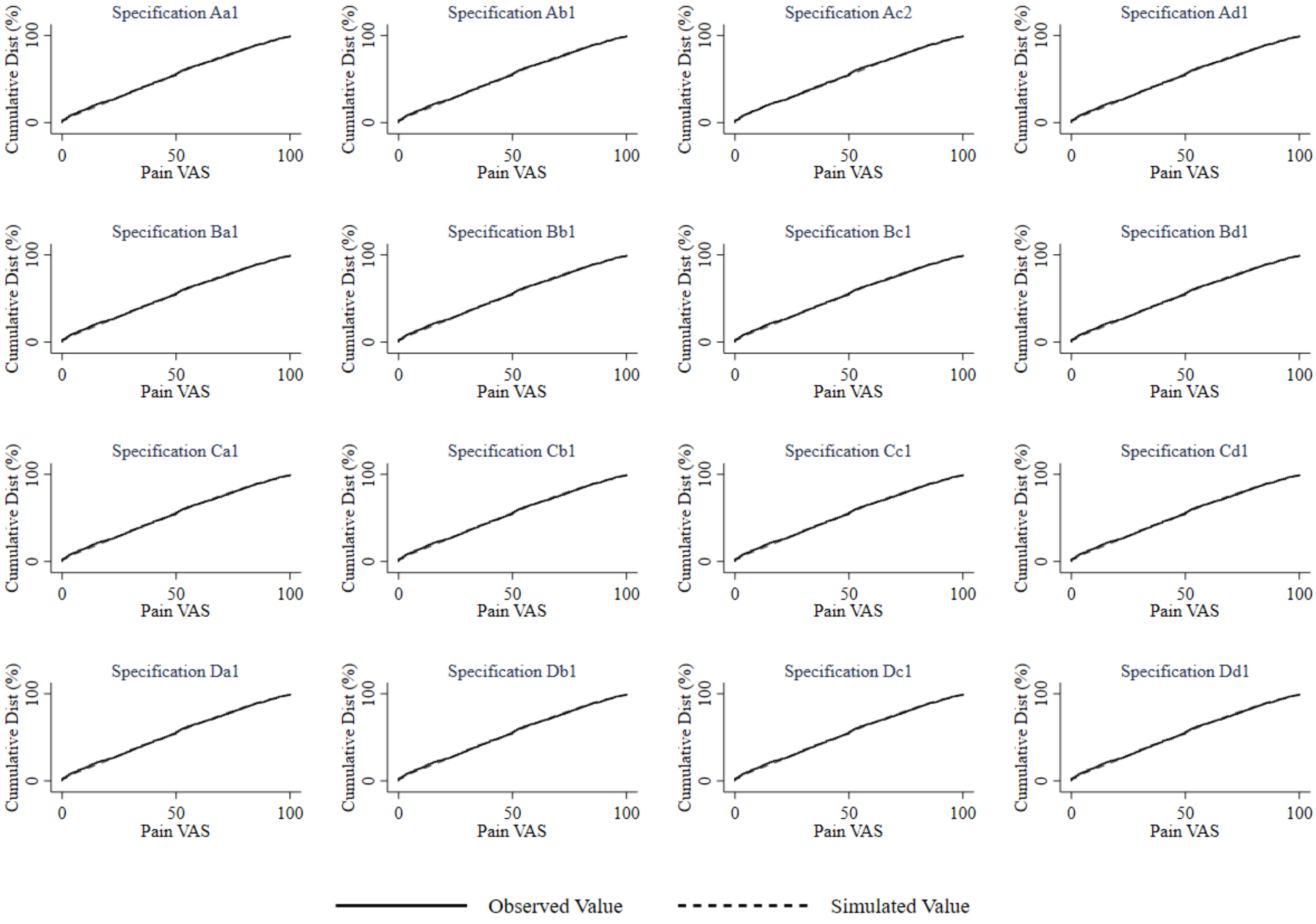

Supplement: Supplementary file 1 — Supplementary file1 (PDF 276 KB) [file 296_2025_5897_MOESM1_ESM.pdf]
